# Cooperative regulation of endocytic vesicle transport by yeast Eps15-like protein Pan1p and epsins

**DOI:** 10.1016/j.jbc.2021.101254

**Published:** 2021-09-27

**Authors:** Nao Yoshida, Ippo Ogura, Makoto Nagano, Tadashi Ando, Junko Y. Toshima, Jiro Toshima

**Affiliations:** 1 Department of Biological Science and Technology, Tokyo University of Science, Katsushika-ku, Tokyo, Japan; 2 Department of Applied Electronics, Tokyo University of Science, Katsushika-ku, Tokyo, Japan; 3 School of Health Science, Tokyo University of Technology, Ota-ku, Tokyo, Japan

**Keywords:** endocytosis, actin, membrane transport, imaging, ACB, actin cytoskeleton-binding, Alexa-α-factor, Alexa Fluor 647–α-factor, CCVs, clathrin-coated vesicles, Ent1ΔACB, deletion of the ACB domain of Ent1p, *pan1-18TA*ΔABD, deletion of the actin-binding domain of Pan1p-18TA, Sla2ΔTHATCH, deletion of the THATCH/talin-like domain of Sla2p, SM, synthetic medium, YPD, yeast extract peptone dextrose

## Abstract

Dynamic actin filaments are required for the formation and internalization of endocytic vesicles. Yeast actin cables serve as a track for the translocation of endocytic vesicles to early endosomes, but the molecular mechanisms regulating the interaction between vesicles and the actin cables remain ambiguous. Previous studies have demonstrated that the yeast Eps15-like protein Pan1p plays an important role in this interaction, and that interaction is not completely lost even after deletion of the Pan1p actin-binding domain, suggesting that additional proteins mediate association of the vesicle with the actin cable. Other candidates for mediating the interaction are endocytic coat proteins Sla2p (yeast Hip1R) and Ent1p/2p (yeast epsins), as these proteins can bind to both the plasma membrane and the actin filament. Here, we investigated the degree of redundancy in the actin-binding activities of Pan1p, Sla2p, and Ent1p/2p involved in the internalization and transport of endocytic vesicles. Expression of the nonphosphorylatable form of Pan1p, Pan1-18TA, caused abnormal accumulation of both actin cables and endocytic vesicles, and this accumulation was additively suppressed by deletion of the actin-binding domains of both Pan1p and Ent1p. Interestingly, deletion of the actin-binding domains of Pan1p and Ent1p in cells lacking the *ENT2* gene resulted in severely defective internalization of endocytic vesicles and recruitment of actin cables to the site of endocytosis. These results suggest that Pan1p and Ent1p/2p cooperatively regulate the interaction between the endocytic vesicle and the actin cable.

Endocytosis is a critical process in which a vesicle containing various cargos, such as extracellular molecules, membrane proteins, or certain types of viruses, buds off from the plasma membrane to internalize its content into the cell. Among the many forms of endocytosis, the best characterized is clathrin-mediated endocytosis (1, 2). In this process, over 50 proteins have been shown to be required for formation and transport of clathrin-coated vesicles (CCVs) (3, 4). Recent live-cell imaging of various eukaryotic cells has revealed that the actin cytoskeleton plays an essential role in the formation and internalization of CCVs (5, 6, 7), and the molecular mechanisms involved have been well characterized. Endocytic vesicles also require actin cables, which are polarized linear bundles of actin filaments (8, 9), to mediate their transport to the early endosome (10, 11), but it still remains unclear how endocytic vesicles interact with such actin cables. Previous studies have demonstrated that endocytic vesicles move on the actin cable, independently of type V myosins (Myo2p and Myo4p), and passively with the cable itself (7, 10). This suggests the existence of endocytic protein(s) that fix the endocytic vesicle to the actin cable through an ability to bind to the vesicle, the cable, or both.

In yeast, after internalization, each CCV is uncoated through the combined actions of phosphatidylinositol 4,5-bisphosphate phosphatases Sjl1p and Sjl2p, Arf3p GTPase, and Ark1p/Prk1p kinases (4, 12). The Ark1p/Prk1p kinases, which are related to the mammalian proteins GAK and AAK1 (13), have been shown to regulate the disassembly of endocytic coat proteins and actin by phosphorylating several target proteins, such as Sla1p, Ent1/2p, Yap1801/2p, Scd5p, and Pan1p (14, 15, 16). A study using a *prk1* analog-sensitive mutant has revealed that transient inactivation of Prk1p causes stable association of endocytic vesicles with actin cables (17, 18), and inhibits dissociation of the vesicle from the cable, suggesting that substrates of Ark1p/Prk1p might be key regulators of endocytic vesicle transport along the actin cable. The Eps15-like protein Pan1p is the major *in vivo* target of Ark1p/Prk1p kinases, and its ability to both bind actin filament and promote actin polymerization is negatively regulated by Prk1p phosphorylation (19). Pan1p contains 18 Ark1p/Prk1p phosphorylation consensus sequences ([L/I/V/M]xx[Q/N/T/S]xTG), and mutation of the 18 threonine to alanine (Pan1p-18TA) causes abnormal cytoplasmic actin aggregates, also called actin clumps, containing endocytic vesicles and endosomes with several endocytic and endosomal proteins (18). The *pan1-18TA* mutant exhibits less-polarized and more-aggregated actin cable structures in the actin clumps (18), indicating that the actin cable might stably associate with the unphosphorylated form of Pan1p, which resides on the endocytic vesicle.

Other candidates for mediating the interaction between the endocytic vesicle and the actin cable are the yeast epsin homologs Ent1p and Ent2p, as these proteins can bind to both the plasma membrane and the actin filament *via* the N-terminal epsin N-terminal homology domain and C-terminal actin-binding domain, respectively (20, 21, 22, 23). Ent1p and Ent2p also have Ark1p/Prk1p phosphorylation consensus sequences. The threonine residues in the consensus sequences are phosphorylated by Prk1p *in vitro*, and the overexpression of the unphosphorylated form of Ent1p partially suppresses the formation of the aberrant actin aggregates observed in *ark1*Δ *prk1*Δ cells (16). In addition, Prk1p phosphorylation sites are located within or in close proximity to the actin cytoskeleton-binding (ACB) domain, and the phospho-mimicking mutation of the Ent1 ACB domain reduces its F-actin-binding activity (22). Considering that Ent1p and Ent2p interact directly with Pan1p (24), the Pan1p–Ent1p complex might play an important role in regulating the interaction between the endocytic vesicle and the actin cable. A previous study has shown that Ent1p and the yeast HIP1R homolog Sla2p interact redundantly with actin filament and that deletion of the actin-binding domains of these proteins causes a severe defect in endocytosis (22). As Sla2p also binds to both the plasma membrane and actin filament *via* the N-terminal ANTH domain and the C-terminal THATCH/talin-like domain (22, 25, 26), Sla2p might also be involved in transport of the endocytic vesicle along the actin cable.

Here, we demonstrate a degree of redundancy in the actin-binding activities of Pan1p, Sla2p, and Ent1p/2p required for the formation and transport of endocytic vesicles. The *pan1* mutant lacking the actin-binding domain was shown to exhibit slightly defective endocytic vesicle internalization when combined with the *sla2* mutant, but markedly defective vesicle internalization as well as recruitment of the actin cable to the endocytic site when combined with the *ent1 ent2* mutant. These results suggest that Pan1p and Ent1p/2p cooperatively regulate the interaction between the endocytic vesicle and the actin cable.

## Results

### Actin-binding domains of Pan1p, Sla2p, and Ent1p/2p are required for the formation of actin cable aggregates in the *pan1-18TA* mutant

**Figure 1:**
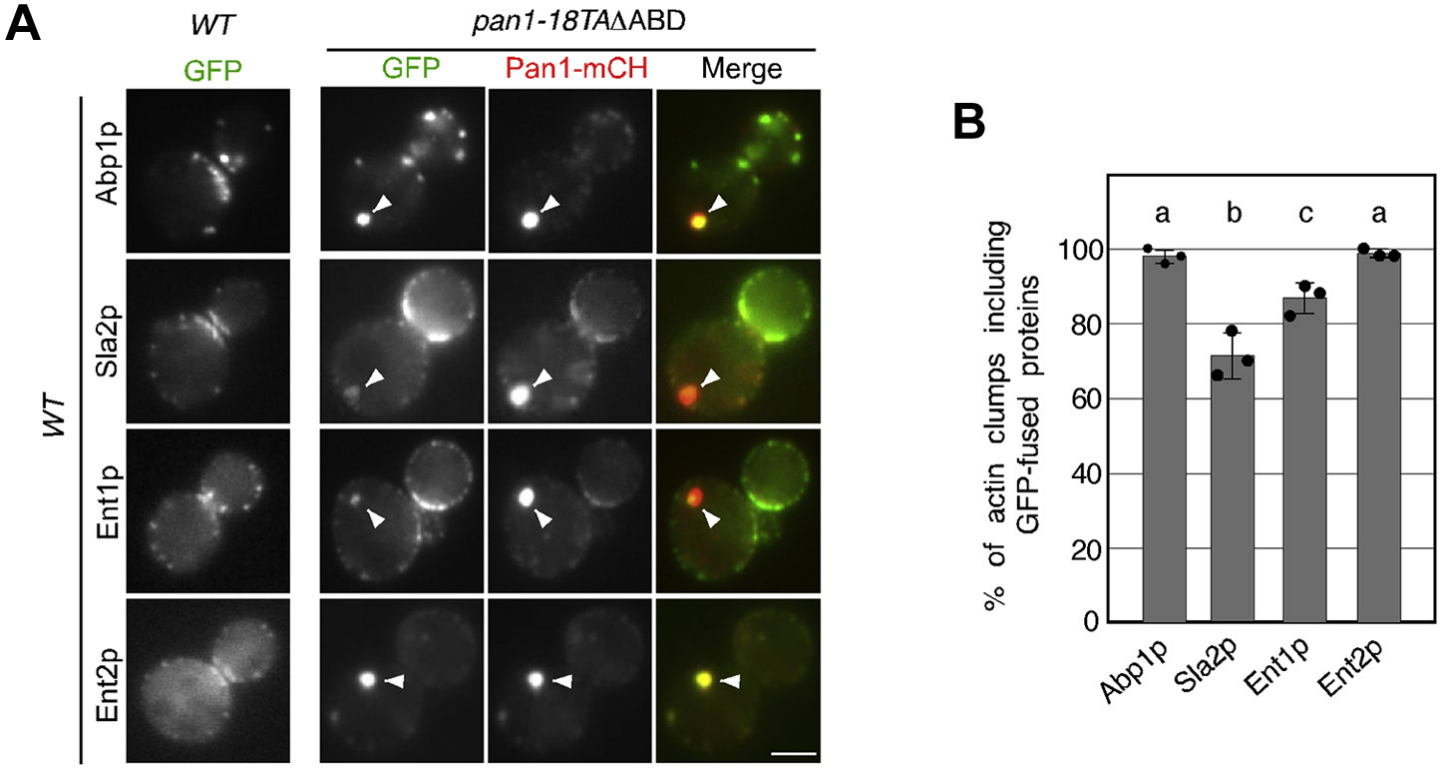
**The localization of Sla2-GFP, Ent1-GFP, and Ent2-GFP in WT and *pan1-18TA* cells.***A*, cells expressing each GFP-tagged protein and Pan1-mCherry were grown to the early logarithmic phase to mid–logarithmic phase in the YPD medium at 25 °C and observed by fluorescence microscopy. Merged images of GFP and mCherry channels are shown in the *right panels*. The scale bar represents 2.5 μm. *B*, quantification of actin clumps including GFP-tagged proteins. The percentages were calculated as the ratio of actin clumps (n = 50) including each protein in each experiment. Error bars indicate the SD from at least three independent experiments. *Different letters* indicate significant difference at *p* < 0.05, one-way ANOVA with Tukey’s post hoc test. YPD, yeast extract peptone dextrose.

**Figure 2:**
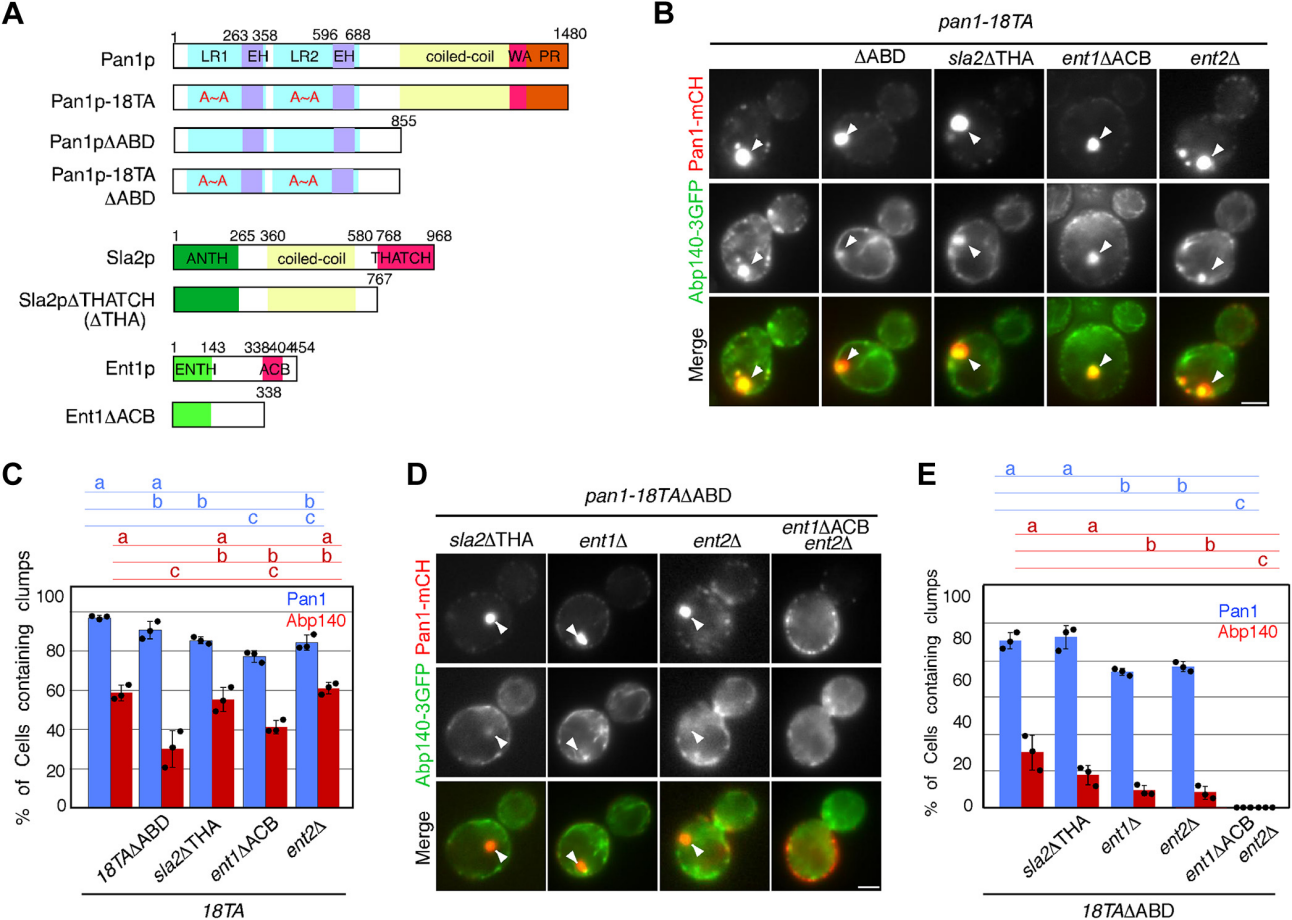
**Redundant role of actin-binding domains of Pan1p, Sla2p, and Ent1p in formation of actin cable aggregates in the *pan1-18TA* mutant.***A*, diagram of Pan1p, Sla2p, and Ent1p constructs used in this study. *B* and *D*, localization of Abp140-3GFP and Pan1-mCherry in *pan1-18TA* (*B*) or *pan1-18TA*ΔABD (*D*) cells lacking indicated protein or domain. *C* and *E*, quantification of cells containing Pan1p or Abp140p clumps. Cells expressing Abp140-3GFP and Pan1-mCherry were grown to the log phase at 25 °C and imaged. Data show the mean ± SD from at least three experiments, with >50 cells counted for each strain per experiment. *Different letters* indicate significant difference at *p* < 0.05, one-way ANOVA with Tukey’s post hoc test (*C* and *E*). The scale bars represent 2.5 μm.

We previously demonstrated that Pan1p directly mediates the interaction between endocytic vesicles and actin cables *via* its actin-binding activity (18). Expression of the Pan1p-18TA mutant causes severe defective endocytosis and abnormal clumping of Pan1p, which contains endocytic vesicles and actin cables (18). Deletion of the actin-binding domain of Pan1p-18TA (*pan1-18TA*ΔABD) decreased the actin cable aggregates, but they were not completely lost in the mutant (18), implying the existence of additional actin-binding coat protein(s) that stabilize vesicle association with actin cables in the clump. A previous study had shown that Sla2p and Ent1p, both of which are mid–coat endocytic proteins, interact redundantly with actin filaments (4, 22). We therefore speculated that Sla2p and/or Ent1p/2p, in addition to Pan1p, might play a role in the interaction between endocytic vesicles and actin cables. To confirm this, we first examined whether Sla2p, Ent1p, and Ent2p are localized to clumps in the *pan1-18TA*Δ*ABD* mutant. We used Abp1-GFP as a marker for actin patch, assumed to be an endocytic vesicle (27), and found that Abp1-GFP is observed in most Pan1p clumps (Fig. 1, *A* and *B*). As expected, the localization of Sla2p, Ent1p, and Ent2p clearly corresponded to that of Pan1p accumulation (Fig. 1*A*). Quantification analysis revealed that Sla2p, Ent1p, or Ent2p was localized at ∼71.3%, ∼86.7%, or 98.7% of Pan1-mCherry–labeled clumps, respectively (Fig. 1*B*). To examine the contribution of the actin-binding activities of these proteins to the formation of actin cable aggregates in the *pan1-18TA* mutant, we constructed the *pan1-18TA* or *pan1-18TA*ΔABD mutant carrying an individual deletion of the THATCH/talin-like domain of Sla2p (Sla2ΔTHATCH) or deletion of the ACB domain of Ent1p (Ent1ΔACB) (Fig. 2*A*). In WT cells, actin cables, labeled by Abp140-3GFP, are highly dynamic polarized structures, whereas those in the *pan1-18TA* mutant were less polarized and had a more-aggregated structure, and ∼58.7% of the mutant contains such actin aggregates (Fig. 2, *B* and *C*). The actin cable aggregates were significantly suppressed in cells expressing the Pan1p-18TA mutant lacking the C-terminal actin-binding domain (*pan1p-18TA*ΔABD), but still remained in ∼30.1% of the cells (Fig. 2, *B* and *C*) (18). The *sla2*ΔTHATCH mutation little affected the actin cable aggregation in the *pan1-18TA* mutant (∼55.3%) (Fig. 2, *B* and *C*), and a combination of the *pan1-18TA*ΔABD and *sla2*ΔTHATCH mutations did not significantly suppress the aggregation of actin cables (∼17.8%), compared with *pan1-18TA*ΔABD mutation (Fig. 2, *D* and *E*). In contrast, the *ent1*ΔACB mutation significantly decreased the actin cable aggregation in the *pan1-18TA* mutant (∼41.4%) (Fig. 2, *B* and *C*), and deletion of the *ENT1* or *ENT2* gene in the *pan1-18TA*ΔABD mutant resulted in a significant decrease of actin cable aggregation (∼9.5% or ∼8.3%, respectively) (Fig. 2, *D* and *E*). Because the functions of Ent1p and En2p partially overlap, and disruption of both genes is lethal (23), we combined *ent1*ΔACB *ent2*Δ mutation with *pan1-18TA*ΔABD mutation to examine the extent to which Ent1p/2p contributes to the actin-binding activity. Intriguingly, in the *pan1-18TA*ΔABD *ent1*ΔABD *ent2*Δ triple mutant, accumulation of Pan1p and actin cables was completely abolished (Fig. 2, *D* and *E*). These results suggest that the actin-binding function of Pan1p and Ent1p/2p might contribute to the interaction between endocytic vesicles and actin cables.

### Pan1p and Ent1p/2p cooperatively regulate actin-dependent endocytic vesicle internalization

**Figure 3:**
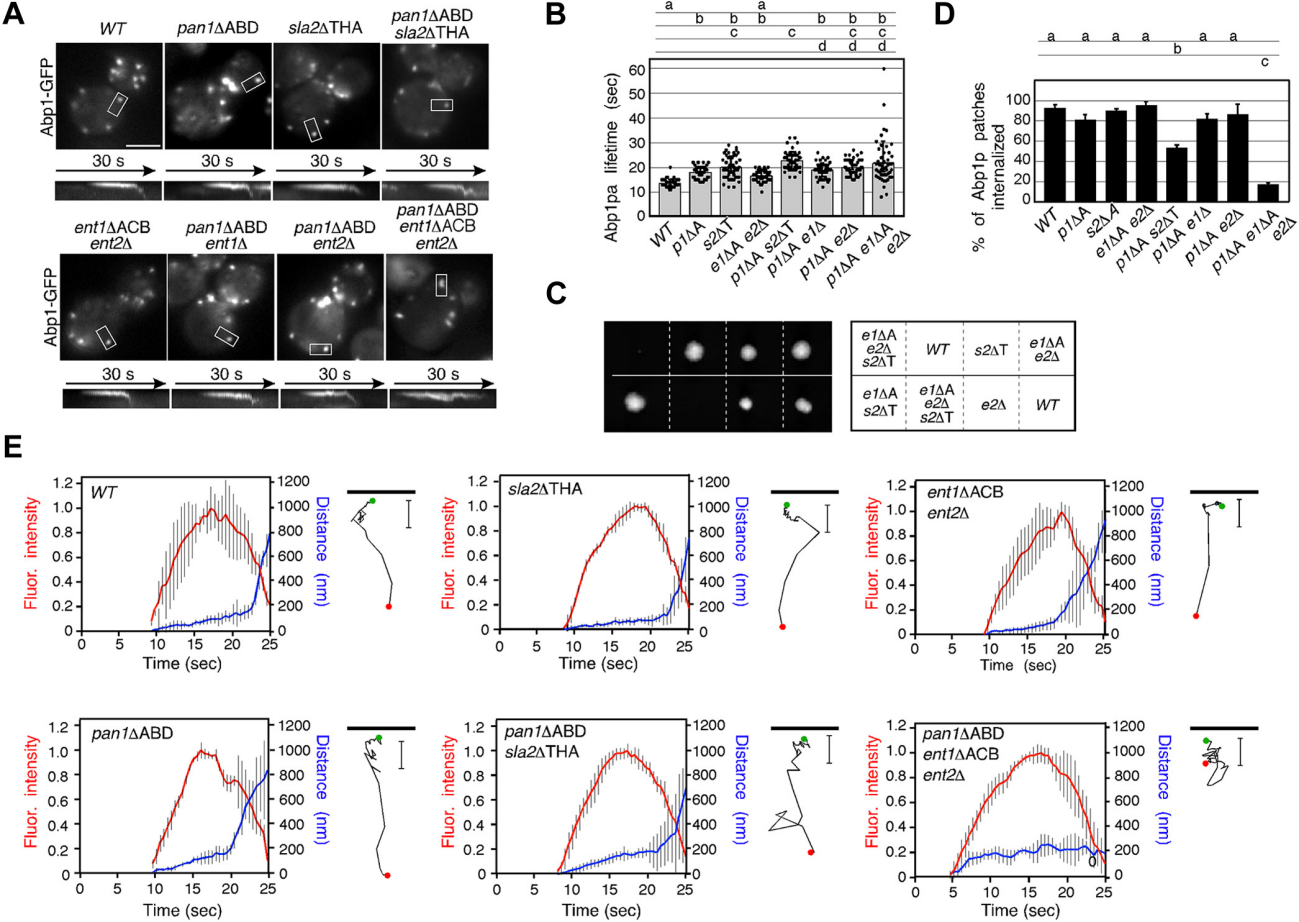
**Redundant role of actin-binding domains of Pan1p, Sla2p, and Ent1p in actin-dependent endocytic vesicle internalization.***A*, localization of Abp1-GFP in indicated mutant cells. Cells expressing Abp1-GFP were grown to the early logarithmic phase to mid–logarithmic phase in the YPD medium at 25 °C and observed by fluorescence microscopy. Kymograph representations of Abp1-GFP from the *boxed area* of strains are indicated in *lower panels*. The scale bars represent 2.5 μm. *B*, average lifetimes of Abp1-GFP patches ± SD in WT and mutant cells. Data were taken from 1-min movies with a 1-s frame interval. n = 50 patches for each strain. *C*, synthetic lethality between *sla2*ΔTHATCH and *ent1*ΔACB *ent2*Δ mutant. Tetrad analysis after sporulation of an *sla2*ΔTHATCH::kan^r^/*SLA2 ent1*ΔACB::kan^r^/*ENT1 ent2*Δ::kan^r^/*ENT2* diploid. The tetrads were dissected on the YPD plate, and the plate was photographed after 3 days of growth at 25 °C. The *right panel* represents the genotype of each spore. *D*, the bar graphs represent the percentage of patches internalized into the cytosol in WT or mutant cells. Data show the mean ± SD from at least three experiments, with 50 patches counted for each strain per experiment. *Different letters* indicate significant difference at *p* < 0.001, one-way ANOVA with Tukey’s post hoc test (*B* and *D*). *E*, quantification of fluorescence intensity (*red*) and distance from the site of patch formation (*blue*) as a function of time for patches of Abp1-GFP. Data from ten patches from each strain were averaged using single-color movies of Abp1-GFP. Fluorescent intensity over time was corrected for photobleaching. Tracking of individual cortical Abp1p patches is indicated in *right*. Abp1-GFP was visualized every 1 s, and patch movement traces were obtained for the entire life of the patches. *Green* and *red dots* indicate the first and the last positions, respectively. YPD, yeast extract peptone dextrose.

We next examined whether lack of the actin-binding activity of these proteins affected the dynamics of endocytic vesicles. The *pan1*ΔABD, *sla2*ΔTHATCH, or *ent1*ΔACB *ent2*Δ mutant exhibited a slightly increased Abp1p patch lifetime (∼17.8 s, ∼20.0 s or ∼16.5 s, respectively) relative to WT cells (∼13.5 s) (Fig. 3, *A* and *B*). The *pan1*ΔABD *ent1*Δ or *pan1*ΔABD *ent2*Δ double mutant also exhibited a slightly increased Abp1p lifetime (∼18.8 s or ∼19.9 s). These effects were clearly enhanced in the *pan1*ΔABD *sla2*ΔTHATCH (∼22.7 s) or *pan1*ΔABD *ent1*ΔACB *ent2*Δ mutant (∼21.9 s) (Fig. 3, *A* and *B*). A previous study had reported that deletion of the actin-binding domains of Sla2p and Ent1p arrested endocytic vesicle budding and that actin was polymerized continuously at sites of endocytosis. We also found that combined deletion of the *ENT2* gene and the actin-binding domains of Sla2p and Ent1p (*sla2*ΔTHATCH *ent1*ΔACB *ent2*Δ mutation) had a lethal outcome (Fig. 3*C*), suggesting an essential role of these actin-binding domains in endocytic vesicle formation. Kymographs and particle tracking analysis showed that the majority of Abp1p patches in the *pan1*ΔABD, *sla2*ΔTHATCH, or *ent1*ΔACB *ent2*Δ mutant were internalized normally (∼81.3%, ∼90.0%, or ∼95.3%, respectively), like WT cells (∼93.3%) (Fig. 3, *A*, *D* and *E*). The *pan1*ΔABD *ent1*Δ or *pan1*ΔABD *ent2*Δ double mutant also exhibited normal Abp1p patch internalization (∼82.0% or ∼86.7%), but the *pan1*ΔABD *sla2*ΔTHATCH and *pan1*ΔABD *ent1*ΔACB *ent2*Δ mutants showed Abp1p patch behaviors distinct from those of the WT cells (Fig. 3, *A*, *D* and *E*). In the *pan1*ΔABD *sla2*ΔTHATCH mutant, the efficiency of Abp1p patch internalization was slightly reduced (∼53.3%) and inward movement from the plasma membrane was irregular, in comparison with the WT cells (Fig. 3, *A*, *D* and *E*). In the *pan1*ΔABD *ent1*ΔACB *ent2*Δ mutant, Abp1p patch dynamics were affected much more severely; the majority of Abp1p patches were not internalized (∼17.3%), and although changes in their fluorescence intensity were similar to those in WT cells, a longer time was required (Fig. 3, *A*, *D* and *E*). When the Abp1p patch increased and reached maximum fluorescence intensity, the *pan1*ΔABD *ent1*ΔACB *ent2*Δ mutant exhibited transient formation of actin tail–like structures that arose from the Pan1-GFP patch, thereafter gradually shortening and disappearing after Pan1p had done so (Fig. 3*A* and Movie S1). These findings suggested that actin polymerization and depolymerization probably occur regularly at endocytic sites but that actin-dependent vesicle internalization is impaired in the *pan1*ΔABD *ent1*ΔACB *ent2*Δ mutant.

### Distinct requirement of actin-binding domains for endocytic internalization

**Figure 4:**
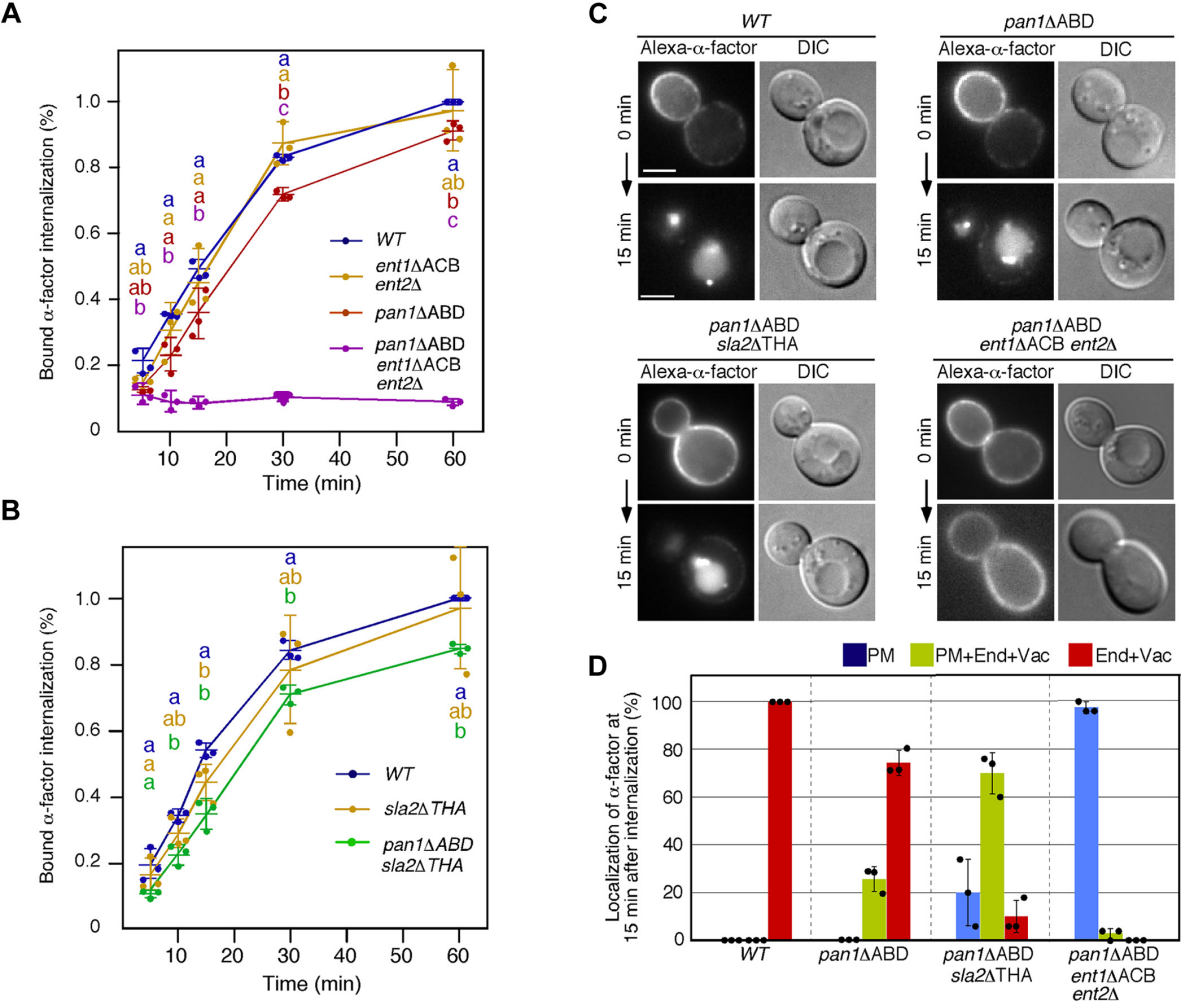
**Effect of deleting each of the actin-binding domains of Pan1p, Sla2p, and Ent1p on the endocytic pathway.***A* and *B*, radiolabeled α-factor internalization assays performed on the indicated strains at 25 °C. Each *curve* represents the average of three independent experiments, and error bars indicate the SD at each time point. *Different letters* indicate significant difference at *p* < 0.05, one-way ANOVA with Tukey’s post hoc test. *C*, transport of Alexa Fluor 647–α-factor in *pan1*ΔABD, *pan1*ΔABD *sla2*ΔTHATCH, or *pan1*ΔABD *ent1*ΔACB *ent2*Δ mutant. WT and mutant cells were treated with Alexa-α-factor, and the images were acquired 0 and 15 min after washing out unbound Alexa-α-factor and warming the cell to 25 °C. The scale bars represent 2.5 μm. *D*, quantification of the localization of Alexa-α-factor in WT and mutant cells at 15 min after internalization. The bar graphs represent the percentages of cells exhibiting Alexa-α-factor localized at the endosome and vacuole (*red*), the plasma membrane, endosome, and vacuole (*yellow*), or the plasma membrane (*blue*). Data show the mean ± SD from three experiments, with 50 cells counted for each strain per experiment. Alexa-α-factor, Alexa Fluor 647–α-factor.

To clarify the step of endocytic transport that requires the actin-binding activities of Pan1p, Sla2p, and Ent1p, we next examined the effect of deleting each of the actin-binding domains on the endocytic pathway. We first carried out an α-factor internalization assay to examine the effect on endocytic cargo internalization. *pan1*ΔABD mutants exhibited only a slight defect of ^35^S-labeled α-factor internalization (Fig. 4*A*, red line), whereas the effect in *ent1*ΔACB *ent2*Δ or *sla2*ΔTHATCH mutants was negligible (Fig. 4, *A* and *B*, yellow lines). Combination of the *pan1*ΔABD and *ent1*Δ*ACB ent2*Δ mutations resulted in a marked defect (Fig. 4*A*, purple line), but a combination of the *pan1*Δ*ABD* mutant with the *sla2*ΔTHATCH mutant resulted in a moderate defect (Fig. 4*B*, green line), consistent with the observation that internalization of Abp1p patches was mostly inhibited in the mutant (Fig. 3*E*).

We next labeled WT and mutant cells with Alexa Fluor 647–α-factor (Alexa-α-factor), a marker of the receptor-mediated endocytic pathway, and followed the localization at 15 min after α-factor internalization to examine the effect on transport of endocytic cargo from the plasma membrane to the vacuole. The *pan1*Δ*ABD* mutant showed a slight delay in α-factor transport to the vacuole (PM only, 0.0%; PM + End + Vac, ∼25.7%; End + Vac, ∼74.3%), but the delay was additive when combined with the *sla2*ΔTHATCH mutant (PM only, ∼10.0%; PM + End + Vac, ∼70.0%; End + Vac, ∼20.0%) (Fig. 4, *C* and *D*). Consistent with the results of analysis using ^35^S-labeled α-factor, the *pan1*ΔABD *ent1*ΔACB *ent2*Δ mutant showed a much severer defect in transport of Alexa-α-factor from the plasma membrane to the cytosol (PM only, ∼97.3%; PM + End + Vac, ∼2.7%; End + Vac, 0.0%) relative to the *pan1*ΔABD *sla2*ΔTHATCH mutant (Fig. 4, *C* and *D*). These results clearly indicated that the *pan1*ΔABD *ent1*ΔACB *ent2*Δ mutant has a defect at the internalization step in the endocytic pathway.

### The actin-binding domains of Pan1p and Ent1/2p are required for recruitment of actin cables to endocytic sites

**Figure 5:**
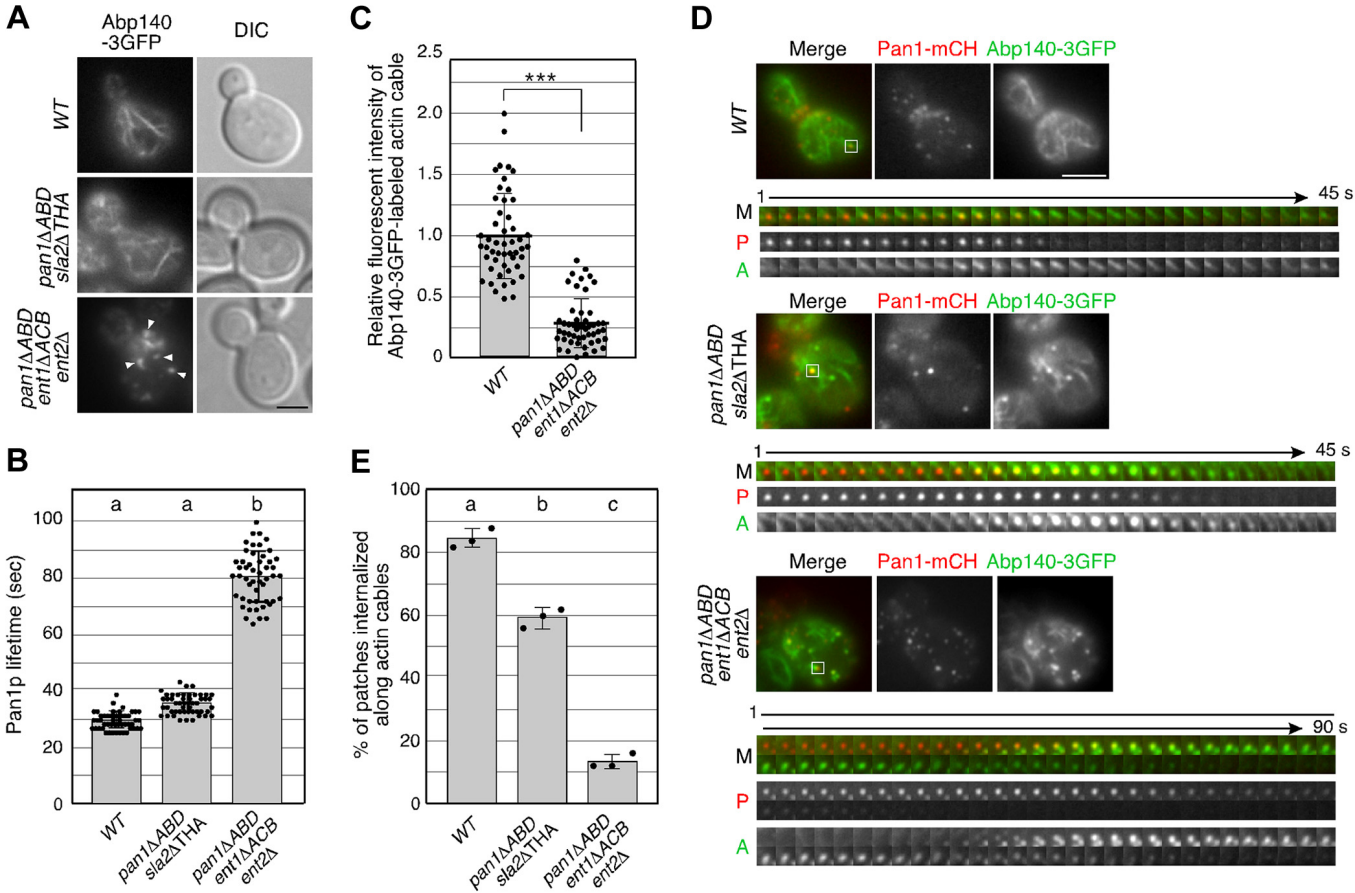
**Interaction between endocytic vesicles and actin cables in WT and mutant cells.***A*, localization of Abp140-3GFP in indicated mutant cells. Cells expressing Abp1-GFP were grown to the early logarithmic phase to mid–logarithmic phase in the YPD medium at 25 °C and observed by fluorescence microscopy and differential interference contrast (DIC). The scale bars represent 2.5 μm. *B*, average lifetimes of Pan1-mCherry patches ± SD in WT and mutant cells. Data were taken from 1- to 3-min movies with a 1-s frame interval. n = 50 patches for each strain. *C*, quantification of the fluorescence intensity of Abp140-3GFP–labeled actin cables in WT and *pan1*ΔABD *ent1*ΔACB *ent2*Δ cells. The fluorescence intensity of actin cables was measured in a randomly selected area (1 × 1 pixel area) on 50 actin cables. For the relative fluorescence intensity, each value was divided by the average fluorescence intensity of actin cables in WT cells. ∗∗∗*p* value <0.001, unpaired *t* test. *D*, the *left images* represent single frames from movies of WT and mutant cells showing GFP (Abp140p) and mCherry (Pan1p) channels, and merged images. Cells expressing Abp140-3GFP and Pan1-mCherry were grown to the log phase at 25 °C, and subsequently imaged at 1.5-s intervals. A time series of single patches in the *boxed area* for each strain is shown in the *lower panels*. The scale bar represents 2.5 μm. *E*, the bar graphs represent the percentage of patches internalized along actin cables in WT or mutant cells. Data show the mean ± SD from three experiments, with >30 patches counted for each strain per experiment. *Different letters* indicate significant difference at *p* < 0.0001, one-way ANOVA with Tukey’s post hoc test (*B* and *C*). YPD, yeast extract peptone dextrose.

We previously showed that over 80% of endocytic vesicles were internalized along actin cables at the internalization step of endocytosis (28). Therefore, we next examined actin cable dynamics in the *pan1*ΔABD *sla2*ΔTHATCH or *pan1*ΔABD *ent1*ΔACB *ent2*Δ mutant. While the *pan1*ΔABD *sla2*ΔTHATCH mutant exhibited a near-normal polarized actin cable structure, the *pan1*ΔABD *ent1*ΔACB *ent2*Δ mutant showed aberrant actin cable dynamics (Fig. 5*A* and Movie S2). The *pan1*ΔABD *sla2*ΔTHATCH mutant exhibited a slightly increased Pan1p patch lifetime (∼35.7 s) relative to WT cells (∼29.7 s) (Fig. 5*B*). In contrast, the *pan1*ΔABD *ent1*ΔACB *ent2*Δ mutant exhibited remarkably increased Pan1p lifetime (∼80.6 s). In the *pan1*ΔABD *ent1*ΔACB *ent2*Δ mutant, particularly, the fluorescence intensity of actin cables, labeled by Abp140-3GFP, was decreased, and instead actin tail–like structures, which were also labeled by Abp1-GFP (Fig. 3*A*), were more clearly evident (Fig. 5, *A* and *C* and Movie S2). Because Abp140p is reported to localize to both actin patches and cables (29), this observation suggests that localization of Abp140-3GFP at actin patches is increased in the mutant because of decrease of actin cables. Most of the actin tail–like structures attached to Pan1-mCherry were transiently assembled and disassembled on the plasma membrane, as described above, but recruitment of actin cables to Pan1-mCherry–labeled endocytic sites and the following Pan1p patch internalization were significantly decreased (∼13.3%) (Fig. 5, *D* and *E*). In the *pan1*ΔABD *sla2*ΔTHATCH mutant, Pan1p patches internalized linearly along actin cables were slightly decreased (∼59.3%), whereas in WT cells, ∼84.7% of them, even in a single focal plane image, were internalized along actin cables (Fig. 5, *D* and *E* and Movie S3). These observations suggested that the actin-binding activities of these three proteins, especially Pan1p and Ent1p/2p, are necessary for the recruitment of actin cables to endocytic vesicles.

## Discussion

**Figure 6:**
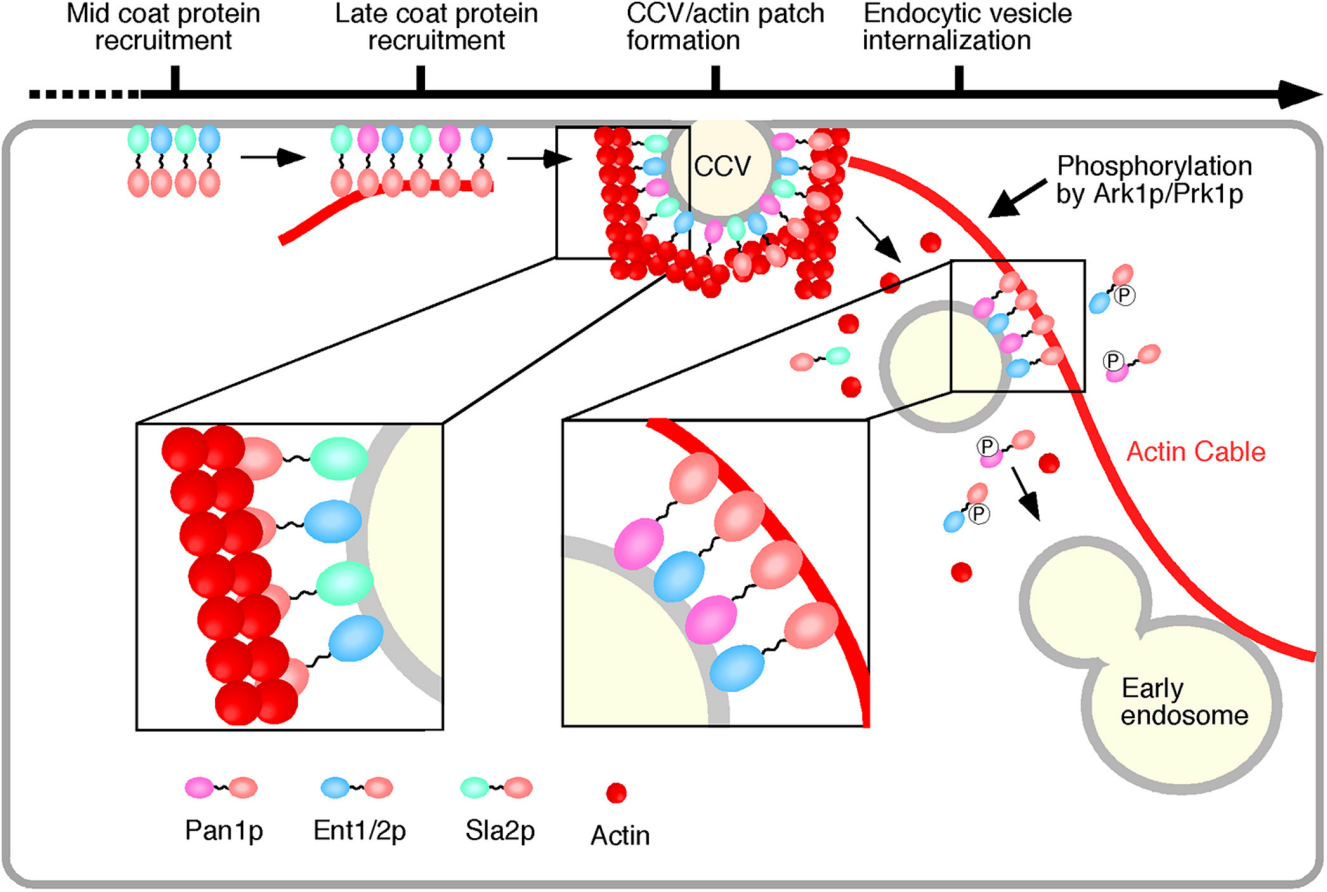
**Model of the actin cable–mediated endocytic pathway.** Unphosphorylated Pan1p and Ent1p/2p on an endocytic vesicle bind to actin to fix the vesicle to the actin cable. After being pinched off from the membrane, the endocytic vesicle moves into the cytosol as a result of actin cable flow (11). Pan1p and Ent1p/2p phosphorylation by Ark1/Prk1 kinases causes dissociation of endocytic vesicle from the actin cable, and then, the vesicle fuses to the early endosome. CCV, clathrin-coated vesicle.

In a previous study, we investigated the timing of actin cable recruitment to sites of endocytosis and demonstrated that ∼48% of actin cables appear at such sites before actin patch formation (28), implying CCV budding, whereas ∼39% of the cables appear thereafter. This observation suggested that actin cables are recruited to endocytic sites independently of CCV budding and that an endocytic protein, which is recruited to the site before vesicle formation and has the ability to bind to the actin filament, mediates the interaction. In yeast endocytosis, early coat proteins, including clathrin and the AP-2 complex, are recruited to the endocytic site first, and then mid–coat proteins, such as Sla2p and Ent1/2p, are recruited around 10 to 20 s before CCV formation (Fig. 6). The Pan1p complex, including Sla1p and End3p, is classified as late coat protein and recruited to the endocytic site slightly after Sla2p and Ent1/2p (4). Considering that the interaction between the endocytic vesicle and the actin cable should be transient and controllable, Pan1p and Ent1/2p are ideal candidates for mediating this interaction. Pan1p can bind to actin filaments directly, and the binding is regulated by phosphorylation through the Ark1p/Prk1p kinases (19). Ent1p and Ent2p are also targets of Ark1/Prk1 kinases (16), and a phospho-mimicking mutant form of the Ent1p ACB domain severely reduces the actin-binding activity (22). As Prk1p and Ark1p are recruited to the endocytic site at 1∼2 s after Abp1p arrival (12), it seems reasonable to assume that–before endocytic vesicle budding–Pan1p and Ent1p/2p recruit actin cables to the endocytic site and are then phosphorylated by Ark1p/Prk1p kinases after vesicle internalization, thereafter dissociating from the actin cable and resulting in separation of the latter from the endocytic vesicle. Endocytic vesicle movement occurs at the same velocity and direction as that of actin cables (11), and this suggests that endocytic vesicles remain fixed on the actin cables and move as a result of actin cable flow. The endocytic vesicle eventually fuses with the early/recycling endosome after these events (Fig. 6).

It has been reported that Pan1p, Sla2p, and Ent1/2p are able to bind actin filaments, but that strains carrying individual deletions of these actin-binding domains exhibit near-normal endocytic dynamics, although the Pan1ΔABD mutation suppresses the formation of actin cable aggregates in the *pant1-18TA* mutant (18, 19, 22, 26). Previous studies have demonstrated that the C-terminal actin-binding domains of Sla2p and Ent1p/2p interact redundantly with actin filaments and that deletion of both actin-binding domains (*sla2*Δ*THATCH ent1*Δ*ACB*) causes a specific endocytic block referred to as the “uncoupling phenotype,” which is also observed in the *sla2*Δ mutant and suggests that the actin cytoskeleton is not coupled to the membrane efficiently (22, 27). The N-terminal lipid-binding domains of Sla2p and Ent1p were shown to coassemble in a PI(4,5)P_2_-dependent manner and form an organized lattice structure, which is required for CCV formation (30). These observations, taken together with our finding that the *sla2*ΔTHATCH *ent1*ΔACB *ent2*Δ triple mutant is nonviable, suggest that the actin-binding domains of Sla2p and Ent1/2p are essential for CCV formation.

In contrast to the *sla2*ΔTHATCH *ent1*ΔACB *ent2*Δ mutant, the *pan1*ΔABD *sla2*ΔTHATCH or *pan1*ΔABD *ent1*ΔABD *ent2*Δ mutant was viable, and both exhibited distinct actin and endocytic phenotypes. While the *pan1*ΔABD *ent1*ΔACB *ent2*Δ mutant exhibited severely defective endocytosis, the *pan1*ΔABD *sla2*ΔTHATCH mutant was only mildly defective in terms of both endocytosis and the actin cytoskeleton, suggesting a distinct role of the actin-binding domains of Pan1p and Sla2p. This idea is also supported by the previous observation that Sla2p specifically inhibits the ability of Pan1p to bind to the actin filament (31). Sla2p binds directly to Pan1p *via* its coiled-coil domain, and a mutant with partial loss of function of *pan1* shows suppression of the endocytic and actin phenotypes observed in the *sla2* mutant lacking the coiled-coil domain (31). Thus, Pan1p might bind to the actin filament after dissociating from Sla2p.

The actin structure observed in the *pan1*ΔABD *ent1*ΔACB *ent2*Δ mutant probably does not correspond to the “uncoupling phenotype” observed in the *sla2*Δ or *sla2*ΔTHATCH *ent1*ΔACB mutant because endocytic coat assembly and disassembly accompanied by actin polymerization and depolymerization occur regularly. Activation of the Arp2/3 complex by nucleation-promoting factors such as Las17p (yeast WASP) and Myo3p/5p (yeast type I myosins) plays an important role in actin patch formation, and these activities are regulated by the endocytic coat protein complex including Pan1p, Sla1p, and End3p (32, 33). In contrast, disassembly of the actin patch is regulated by phosphorylation of coat proteins by Ark1p/Prk1p kinases (13). In the *pan1*ΔABD *ent1*ΔACB *ent2*Δ mutant, actin patch internalization was severely blocked, whereas the lifetime of actin patch was increased only slightly, suggesting that the vesicle budding step mediated by the interaction between endocytic coat proteins and actin regulators is normal; however, the vesicle scission or internalization step is impaired. The interaction between the endocytic vesicle and the actin cable may play a role in these steps, as well as transport to the endosome.

Our results suggest the important role of Pan1p and Ent1/2p in the interaction between the endocytic vesicle and the actin cable, but the role of clathrin in the interaction remains unclear. Previous studies have demonstrated that deletion of the *CHC1* or *CLC1* gene, encoding yeast clathrin heavy chain or light chain, dramatically reduces the inward movement of coat proteins that implies the formation of endocytic vesicle, whereas internalization of actin patches is not inhibited (34, 35). It has been also reported that the timing of actin cable recruitment to actin patches in both *chc1*Δ and *clc1*Δ mutants is similar to that in WT cells (28). These observations suggest that clathrin is required for the link between the coat proteins and actin for endocytic vesicle formation, rather than for the fixing of endocytic vesicle to the actin cable.

## Experimental procedures

### Yeast strains, growth conditions, and plasmids

The yeast strains used in this study are listed in the strain list (Table S1). All strains were grown in yeast extract peptone dextrose (YPD) or synthetic medium (SM) supplemented with 2% glucose and appropriate amino acids. C-terminal GFP or mCherry tagging of proteins was performed by PCR-based homologous recombination using pFA6a-GFP(S65T)-HIS3 (36) or pFA6a-mCherry-URA3 (37), respectively, as a template. The *pan1-18TA* and *sla2*ΔTHATCH mutants were generated as described previously (18, 26). The *pan1*Δ*ABD* or *ent1*ΔACB mutant was generated by PCR-based homologous recombination. To generate the *pan1*ΔABD mutant, the mCherry-URA3 fragment containing 50-bp flanking homologous regions (nt 2513–2562 and the 3′ noncoding region of *PAN1* gene) was amplified using primers (JT2676 and JK2) and pFA6a-mCherry-URA3 as a template and transformed into WT cells. The *pan1*ΔABD mutants were selected on the synthetic complete plate lacking uracil and confirmed by PCR using JT3 and JT2769. To generate the *ent1*ΔACB mutant, the 3HA-HIS3 fragment containing 50-bp flanking homologous regions (nt 965–1014 and the 3′ noncoding region of *ENT1* gene) was amplified using primers (JT2874 and JT2875) and pFA6a-3HA-HIS3 as a template and transformed into WT cells. The *ent1*ΔACB mutants were selected on the synthetic complete plate lacking histidine and confirmed by PCR using JT2876 and JT763.

### Fluorescence microscopy

Fluorescence microscopy was performed using an Olympus IX81 microscope equipped with a 100×/NA 1.40 (Olympus) objective and Orca-AG cooled CCD camera (Hamamatsu), using MetaMorph software (Universal Imaging). Simultaneous imaging of red and green fluorescence was performed using an Olympus IX81 microscope, described above, and an image splitter (Dual-View; Optical Insights) that divided the red and green components of the images with a 565-nm dichroic mirror and passed the red component through a 630/50 nm filter and the green component through a 530/30 nm filter. These split signals were taken simultaneously with one CCD camera, described above.

### Fluorescent labeling of the α-factor and endocytosis assays

Fluorescent labeling of the α-factor was performed as described previously (10). For endocytosis assays, cells were grown to an A_600_ of ∼0.5 in 0.5-ml YPD, briefly centrifuged, and resuspended in 20-μl SM with 5 μM Alexa-α-factor. After incubation on ice for 2 h, the cells were washed with ice-cold SM. Internalization was initiated by addition of SM containing 4% glucose and amino acids at 25 °C.

### ^35^S-labeled α-factor internalization assay

Preparation and internalization of ^35^S-labeled α-factor was performed as described previously (19). Briefly, cells were grown to an OD600 of 0.3 in 50-ml YPD, briefly centrifuged, and resuspended in 4-ml YPD containing 1% (w/v) bovine serum albumin, 50 mM KH_2_PO_4_, pH 6.0, and 20 μg/ml uracil, adenine, and histidine. After adding ^35^S-labeled α-factor, cell aliquots were withdrawn at various time points and subjected to a wash in pH 1 buffer to remove surface-bound α-factors so that internal α-factors could be measured, or in pH 6 buffer to determine the total (internal and bound) α-factors. The amount of cell-associated radioactivity after each wash was determined by scintillation counting. Each experiment was performed at least three times.

## Data availability

All data generated and analyzed during this study are contained within the article and can be shared upon request (jtosiscb@rs.tus.ac.jp).

## Supporting information

This article contains supporting information.

## Conflict of interest

The authors declare that they have no conflicts of interest with the contents of this article.

## Supporting information

Movie S3

Video Captions
