# Supplementary material for: Cooperative regulation of endocytic vesicle transport by yeast Eps15-like protein Pan1p and epsins
Source: J Biol Chem. 2021 Sep 27;297(5):101254. doi: 10.1016/j.jbc.2021.101254 (PMC8628263; doi:10.1016/j.jbc.2021.101254)
Supplement: Supplemental Table S1 [file mmc1.docx]

**Table S1.** Yeast Strains

Strain Genotype Source

JJTY0132 *Mat*α *his3*-Δ*200* *leu2-3*, *112* *ura3-52 lys2-801 ENT1-GFP*::*HIS3* Toshima lab

JJTY0370 *Mat***a** *his3*-Δ*200* *leu2-3*, *112* *ura3-52* *lys2-801 ABP1-GFP*::*HIS3* Toshima lab

JJTY0828 *Mat***a** *his3*-Δ*200* *leu2-3*, *112* *ura3-52* *lys2-801* *SLA2-GFP*::*HIS3 bar1*Δ::*LEU2* Toshima lab

JJTY1936 *Mat*α *his3*-Δ*200* *leu2-3*, *112* *ura3-52 lys2-801 ENT2-GFP*::*HIS3* Toshima lab

JJTY5891 *Mat***a** *his3*-Δ*200, leu2-3*, *112* *ura3-52 lys2-801 pan1*Δ::*pan1-18TA*(*LEU2*)*ΔABD-mCherry*::*URA3 ABP1-GFP*::*HIS3* This Study

JJTY5895 *Mat***a** *his3*-Δ*200, leu2-3*, *112* *ura3-52 lys2-801 pan1*Δ::*pan1-18TA*(*LEU2*)*ΔABD-mCherry*::*URA3 ENT1-GFP*::*HIS3* This Study

JJTY6095 *Mat***a** *his3*-Δ*200, leu2-3*, *112* *ura3-52 lys2-801 pan1*Δ::*pan1-18TA*(*LEU2*)*ΔABD-mCherry*::*URA3 ENT2-GFP*::*HIS3* This Study

JJTY6096 *Mat***a** *his3*-Δ*200, leu2-3*, *112* *ura3-52 lys2-801 pan1*Δ::*pan1-18TA*(*LEU2*)*ΔABD-mCherry*::*URA3 SLA2-GFP*::*HIS3* This Study

JJTY7309 *Mat***a** *his3*-Δ*200, leu2-3*, *112* *ura3-52 lys2-801 pan1*Δ::*pan1-18TA*(*LEU2*)*-mCherry*::*URA3 ABP140-3GFP*::*HIS3* This Study

JJTY7310 *Mat***a** *his3*-Δ*200, leu2-3*, *112* *ura3-52 lys2-801 pan1*Δ::*pan1-18TA*(*LEU2*)*ΔABD-mCherry*::*URA3*

*ABP140-3GFP*::*HIS3* This Study

JJTY8088 *Mat***a** *his3*-Δ*200, leu2-3*, *112* *ura3-52 lys2-801 pan1*Δ::*pan1-18TA*(*LEU2*)*-mCherry*::*URA3 ABP140-3GFP*::*HIS3*

*ent2*Δ::*HIS3* This Study

JJTY10312 *Mat***a** *his3*-Δ*200, leu2-3*, *112* *ura3-52 lys2-801 pan1*Δ::*pan1-18TA*(*LEU2*)*-mCherry*::*URA3 ABP140-3GFP*::*HIS3*

*ent1*Δ*ACB*::*KanMX* This Study

JJTY10313 *Mat***a** *his3*-Δ*200, leu2-3*, *112* *ura3-52 lys2-801 pan1*Δ::*pan1-18TA*(*LEU2*)*-mCherry*::*URA3 ABP140-3GFP*::*HIS3*

*sla2*Δ*THATCH*::*KanMX* This Study

JJTY6103 *Mat***a** *his3*-Δ*200, leu2-3*, *112* *ura3-52 lys2-801* *pan1*Δ::*pan1-18TA*(*LEU2*)*ΔABD-mCherry*::*URA3 Abp140-3GFP*::*HIS3*

*sla2*Δ*THATCH*::*URA3* This Study

JJTY7311 *Mat***α** *his3*-Δ*200, leu2-3*, *112* *ura3-52 lys2-801 pan1*Δ::*pan1-18TA*(*LEU2*)*ΔABD-mCherry*::*URA3 Abp140-3GFP*::*HIS3*

*ent2*Δ::*HIS3* This Study

JJTY8062 *Mat***a** *his3*-Δ*200, leu2-3*, *112* *ura3-52 lys2-801 pan1*Δ::*pan1-18TA*(*LEU2*)*ΔABD-mCherry*::*URA3 Abp140-3GFP*::*HIS3*

*ent1*Δ*::HIS3* This Study

JJTY7751 *Mat***a** *his3*-Δ*200* *leu2-3*, *112* *ura3-52* *bar1*Δ::*LEU2 pan1*Δ::*pan1-18TA*(*LEU2*)*ΔABD-mCherry*::*URA3*

*ABP140-3GFP*::*HIS3 ent1*Δ*ACB::HIS3 ent2Δ::URA3* This Study

JJTY6989 *Mat***α ***his3*-Δ*200* *leu2-3*, *112* *ura3-52* *bar1*Δ::*LEU2 pan1*Δ*ABD-mCherry*::*URA3 ABP1-GFP*::*HIS3*

*sla2*Δ*THATCH*::*URA3* This Study

JJTY6990 *Mat***a** *his3*-Δ*200* *leu2-3*, *112* *ura3-52* *bar1*Δ::*LEU2 PAN1-mCherry*::*URA3 ABP1-GFP*::*HIS3 sla2*Δ*THATCH*::*URA3* This Study

JJTY6991 *Mat***a** *his3*-Δ*200* *leu2-3*, *112* *ura3-52* *lys2-801 PAN1-mCherry*::*URA3 ABP1-GFP*::*HIS3* This Study

JJTY6992 *Mat***a** *his3*-Δ*200* *leu2-3*, *112* *ura3-52* *lys2-801 pan1*Δ*ABD-mCherry*::*URA3 ABP1-GFP*::*HIS3* This Study JJTY7737 *Mat***α** *his3*-Δ*200* *leu2-3*, *112* *ura3-52 lys2-801 pan1ΔABD-mCherry*::*URA3 ABP1-GFP*::*KanMX ent1*Δ::*HIS3* This Study

JJTY7739 *Mat***α** *his3*-Δ*200* *leu2-3*, *112* *ura3-52 lys2-801 pan1ΔABD-mCherry*::*URA3 ABP1-GFP*::*KanMX ent2*Δ::*HIS3* This Study

JJTY7746 *Mat***α** *his3*-Δ*200* *leu2-3*, *112* *ura3-52 lys2-801 PAN1-mCherry*::*LEU2 ABP1-GFP*::*KanMX6 ent1*Δ*ACB*::*HIS3*

*ent2Δ*::*URA3* This Study

JJTY7750 *Mat***α ***his3*-Δ*200* *leu2-3*, *112* *ura3-52 lys2-801 pan1ΔABD-mCherry*::*URA3 ABP1-GFP*::*KanMX6 ent1*Δ*ACB*::*HIS3 ent2Δ*::*URA bar1*Δ::*LEU2* This Study

JJTY8072 *Mat***a** *his3*-Δ*200* *leu2-3*, *112* *ura3-52 lys2-801 ABP140-3GFP*::*HIS3 ABP1-GFP*::*KanMX ent1*Δ*ACB*::*HIS3*

*ent2Δ::URA3 bar1Δ*::*LEU2* This Study

JJTY9499 *Mat***a** *his3*-Δ*200* *leu2-3*, *112* *ura3-52 lys2-801 pan1ΔABD-mCherry*::*URA3 ABP140-3GFP*::*HIS3 ent1*Δ*ACB*::*HIS3*

*ent2Δ*::*URA3 bar1*Δ::*LEU2* This Study

JJTY6983 *Mat***a** *his3*-Δ*200* *leu2-3*, *112* *ura3-52* *bar1*Δ::*LEU2 PAN1-mCherry*::*URA3 ABP140-3GFP*::*HIS3* This Study

JJTY6986 *Mat***a** *his3*-Δ*200* *leu2-3*, *112* *ura3-52* *bar1*Δ::*LEU2 PAN1-mCherry*::*URA3 ABP140-3GFP*::*HIS3*

*sla2*Δ*THATCH*::*URA3* This Study

JJTY6238 *Mat***a** *his3*-Δ*200* *leu2-3*, *112* *ura3-52* *bar1*Δ::*LEU2 pan1*Δ*ABD-mCherry*::*URA3 ABP140-3GFP*::*HIS3* This Study

JJTY6984 *Mat***a** *his3*-Δ*200* *leu2-3*, *112* *ura3-52* *bar1*Δ::*LEU2 pan1*Δ*ABD-mCherry*::*URA3ABP140-3GFP*::*HIS3*

*sla2*Δ*THATCH*::*URA3* This Study
