# Supplementary material for: Cooperative regulation of endocytic vesicle transport by yeast Eps15-like protein Pan1p and epsins
Source: J Biol Chem. 2021 Sep 27;297(5):101254. doi: 10.1016/j.jbc.2021.101254 (PMC8628263; doi:10.1016/j.jbc.2021.101254)
Supplement: Video Captions [file mmc5.docx]

**Movie S1**

Dynamics of Abp1-GFP in wild-type (left) and *pan1*ΔABD *ent1*ΔACB *ent2*Δ mutant cells (right). Arrowheads indicate example of Abp1-GFP movement in each cell. Interval between frames is 0.5 sec.

**Movie S2**

Dynamics of Abp140-3GFP in wild-type (left), *pan1*ΔABD *sla2*ΔTHATCH, (center) and *pan1*ΔABD *ent1*ΔACB *ent2*Δ mutant cells (right). Arrowheads indicate example of Abp140-3GFP-labeled actin-tail structure. Interval between frames is 1.5 sec.

**Movie S3**

Dynamics of Pan1-mCherry (red in merge) and Abp140-3GFP (green in merge) in wild-type (upper images), *pan1*ΔABD *sla2*ΔTHATCH, (center images) and *pan1*ΔABD *ent1*ΔACB *ent2*Δ mutant cells (right images). Arrowheads indicate movement of Abp1-mCherry-labeled endocytic vesicle. Interval between frames is 1.5 sec.
